# A lysin motif effector subverts chitin‐triggered immunity to facilitate arbuscular mycorrhizal symbiosis

**DOI:** 10.1111/nph.16245

**Published:** 2019-11-23

**Authors:** Tian Zeng, Luis Rodriguez‐Moreno, Artem Mansurkhodzaev, Peng Wang, Willy van den Berg, Virginie Gasciolli, Sylvain Cottaz, Sébastien Fort, Bart P. H. J. Thomma, Jean‐Jacques Bono, Ton Bisseling, Erik Limpens

**Affiliations:** ^1^ Laboratory of Molecular Biology Wageningen University & Research 6708 PB Wageningen the Netherlands; ^2^ Department of Plant Sciences Laboratory of Phytopathology Wageningen University & Research 6708 PB Wageningen the Netherlands; ^3^ Laboratory of Biochemistry Wageningen University & Research 6708 WE Wageningen the Netherlands; ^4^ LIPM Université de Toulouse INRA CNRS Castanet‐Tolosan France; ^5^ CNRS CERMAV University Grenoble Alpes UPR 5301 38041 Grenoble France

**Keywords:** arbuscular mycorrhiza, chitin, effector, LysM, plant immunity, symbiosis

## Abstract

- Arbuscular mycorrhizal (AM) fungi greatly improve mineral uptake by host plants in nutrient‐depleted soil and can intracellularly colonize root cortex cells in the vast majority of higher plants. However, AM fungi possess common fungal cell wall components such as chitin that can be recognized by plant chitin receptors to trigger immune responses, raising the question as to how AM fungi effectively evade chitin‐triggered immune responses during symbiosis.
- In this study, we characterize a secreted lysin motif (LysM) effector identified from the model AM fungal species *Rhizophagus irregularis*, called RiSLM.
- RiSLM is one of the highest expressed effector proteins in intraradical mycelium during the symbiosis. *In vitro* binding assays show that RiSLM binds chitin‐oligosaccharides and can protect fungal cell walls from chitinases. Moreover, RiSLM efficiently interferes with chitin‐triggered immune responses, such as defence gene induction and reactive oxygen species production in *Medicago truncatula.* Although RiSLM also binds to symbiotic (lipo)chitooligosaccharides it does not interfere significantly with symbiotic signalling in *Medicago*. Host‐induced gene silencing of *RiSLM* greatly reduces fungal colonization levels.
- Taken together, our results reveal a key role for AM fungal LysM effectors to subvert chitin‐triggered immunity in symbiosis, pointing to a common role for LysM effectors in both symbiotic and pathogenic fungi.

## Introduction

Plants interact with microbes that can range from pathogenic to mutualistic. Typically, plants sense the presence of such microbes by the perception of microbe‐associated molecular patterns (MAMPs) that are recognized by plant pattern recognition receptors. A major MAMP is chitin, a homopolymer of unbranched β‐1,4‐linked *N*‐acetylglucosamine that is a key component of fungal cell walls (Sánchez‐Vallet *et al.*, 2015). In particular, its breakdown products, chitooligosaccharides (COs), are perceived by plant lysin motif (LysM) plasma membrane receptors that trigger immune responses such as defence‐gene induction, secretion of chitinases, and generation of reactive oxygen species (ROS; Miya *et al.*, 2007; Shimizu *et al.*, 2010; Bozsoki *et al.*, 2017). Nevertheless biotrophic pathogenic fungi that colonize plants have evolved strategies to evade or downregulate chitin‐triggered immune responses, especially by secreting specific effectors (Rovenich *et al.*, 2016).

One strategy relies on the secretion of effector proteins that interfere with chitin‐triggered immunity (Sánchez‐Vallet *et al.*, 2015; Rovenich *et al.*, 2016). These include secreted proteins that consist of one to three LysM domains (Kombrink & Thomma, 2013). A key example is the LysM effector Ecp6 from *Cladosporium fulvum*, which is able to sequester fungal cell‐wall‐derived COs (de Jonge *et al.*, 2010). LysM effectors have since been identified in several foliar pathogenic fungi, including the wheat pathogen *Zymoseptoria tritici* (*Mycosphaerella graminicola*; Mg1LysM and Mg3LysM), the rice blast fungus *Magnaporthe oryzae* (Slp1), the Brassicaceae anthracnose fungus *Colletotrichum higginsianum* (ChELP1 and 2), and in the generalist vascular wilt fungal pathogen *Verticillium dahliae* (Marshall *et al.*, 2011; Mentlak *et al.*, 2012; Lee *et al.*, 2014; Takahara *et al.*, 2016; Kombrink *et al.*, 2017). Such LysM effectors can protect fungal hyphae against the activity of plant chitinases (Mg1LysM and Mg3LysM) or suppress chitin‐triggered defence responses by sequestering chitin fragments (Elp1, Ecp6) or have both activities (Mg3LysM) (Sánchez‐Vallet *et al.*, 2015; Rovenich *et al.*, 2016). The occurrence of LysM effectors in such a variety of pathogenic fungi suggests they may be a widespread strategy for fungi to subvert chitin‐triggered immunity in a broad range of hosts.

Mutualistic arbuscular mycorrhizal (AM) fungi establish an intimate interaction with the vast majority (> 80%) of all higher plants (Smith & Read, 2008). During this symbiosis, the fungi are intracellularly hosted in the inner root cortical cells, forming highly branched hyphal structures, called arbuscules (ARBs), where nutrients are exchanged in a cooperative manner (Gutjahr & Parniske, 2013). Chitin is also a key component of the cell wall of AM fungi, and consistently it has been reported that plant defence responses are induced in the early stage of AM symbiosis, but these are quickly suppressed as the interaction progresses (Gianinazzi‐Pearson *et al.*, 1996; García‐Garrido & Ocampo, 2002; Zamioudis & Pieterse, 2012). How AM fungi suppress chitin‐induced immune responses is not known. Here, we study whether AM fungi use similar effectors as pathogenic fungi to suppress this response.

Genomic analyses have identified a LysM domain containing effector (RiSLM; SECRETED LYSM protein) in the genome of the AM fungus *Rhizophagus irregularis* DAOM197198 (Tisserant *et al.*, 2013; Sędzielewska Toro & Brachmann, 2016; Zeng *et al.*, 2018; Schmitz *et al.*, 2019). A homologue of this LysM effector, consisting of a single LysM domain, is conserved in a wide range of AM fungal species, and it was recently shown to be able to bind both chitin and chitosan (Schmitz *et al.*, 2019). However, its role in the symbiosis was not investigated. Here, we show that RiSLM is one of the most highly expressed effectors in the interaction with an evolutionary diverse range of AM host plants. *RiSLM* is induced by strigolactones/root exudates and most highly expressed in the intraradical mycelium (IRM) in *Medicago*. Our binding studies show that RiSLM can bind COs in the low micromolar range, as well as lipo‐COs (LCOs) in the nanomolar range. We show that RiSLM can protect fungal hyphae from plant chitinases and that it efficiently interferes with chitin‐triggered immune responses in the plant. Host‐induced gene silencing of *RiSLM* greatly reduces fungal infection and ARB abundance in the host. Taken together, our results indicate that, in analogy to pathogenic fungi, AM fungi use LysM effectors to suppress chitin‐triggered immunity to mediate a successful symbiosis.

## Materials and Methods

### Protein purification

The coding sequence of RiSLM without signal peptide was amplified using Phusion DNA polymerase (Thermo Fisher Scientific, Waltham, MA, USA). *Eco*RI and *Hin*dIII restriction sites were added to the primers (Supporting Information Table S1) to facilitate cloning into the pET‐SUMO vector (Champion™ pET SUMO Expression System). The amplified RiSLM coding sequence (CDS) was first cloned into the CloneJET vector (Thermo Fisher Scientific) and subsequently cloned *Eco*RI‐*Hin*dIII into the pET‐SUMO vector. All constructs were verified by Sanger sequencing. The pET‐SUMO vector containing RiSLM was transformed into *Escherichia coli* electrocompetent ORIGAMI cells (genetically modified to allow disulphide bond formation). The bacteria were incubated in LB medium supplemented with 50 µg ml^−1^ kanamycin, 10 mM potassium dihydrogen phosphate (KH_2_PO_4_), 50 mM magnesium sulphate and 1% glycerol until an OD_600_ of 0.8 at 28°C. After that, isopropyl β‐d‐1‐thiogalactopyranoside was added at a concentration of 0.05 mM into the culture and incubated at 28°C overnight. Bacterial cultures were then pelleted and stored at −80°C.

For protein purification, frozen bacteria were resuspended in 20 ml lysis buffer (50 mM Tris hydrochloride (Tris‐HCl), 150 mM sodium chloride (NaCl), 10% glycerol, 120 mg lysozyme, 40 mg sodium deoxycholate, 1.25 mg DNAse and one protease cocktail) per litre culture followed by incubation on ice for 2 h. The lysate was transferred into 50 ml tubes and centrifuged for 1 h at 22 000 ***g*** at 4°C. The supernatant was collected and the SUMO‐tagged LysM protein was purified by passing through a BioLogic low‐pressure chromatography system using an NTA Superflow metal affinity chromatography column (Qiagen) according to the manufacturer’s instructions. Eluted protein was incubated in 200 mM NaCl in a dialysis membrane with ULP‐1 protease to remove the SUMO tag overnight at 4°C. After dialysis, the sample was passed again through the NTA column and further purified by size exclusion chromatography using a Superdex 75 on an ÄKTA fast‐performance liquid chromatograph (GE Healthcare, Eindhoven, the Netherlands) using 50 mM Tris‐HCl, 150 mM NaCl (pH 7.4) as elution buffer. Final purified protein was concentrated using an Amicon Ultra‐15 centrifugal device (EMD Millipore, Amsterdam, the Netherlands), and the concentration was determined by Qubit protein assay kit (Thermo Fisher Scientific).

### Chitin binding affinity precipitation assays

Purified protein was incubated with 5 mg of insoluble chitin, chitosan, cellulose, or xylan (Sigma‐Aldrich, Houten, the Netherlands) in 800 μl (1 μM final concentration of RiSLM) of chitin binding buffer (50 mM Tris‐HCl pH 8.0, 150 mM NaCl) at room temperature for 3 h. The tubes were then spun down for 5 min at 1800 ***g*** and the supernatant was removed. The resulting pellet was washed three times with 500 μl of chitin binding buffer and resuspended in 200 μl of sodium dodecyl sulphate (SDS) loading buffer. The supernatants were concentrated to 250 μl, and then 18 μl of supernatant was mixed with 6 μl of 4× SDS sample buffer. The pellets and supernatant in SDS were boiled at 95°C for 5 min before loading for polyacrylamide gel electrophoresis. Proteins gels were stained by standard Coomassie brilliant blue R‐250 staining.

### Microscale thermophoresis

Binding experiments were performed by microscale thermophoresis with a Monolith NT.115 (NanoTemper Technologies, Munich, Germany). RiSLM was labelled with the Monolith NT™ Protein Labeling Kit RED according to the instructions provided by the manufacturer, using a 1 : 3 protein : dye molar ratio. For CO binding experiments, the labelled protein (20 nM) was incubated with a range of titrant concentrations made by serial dilutions (1 : 1), in 50 mM Tris buffer pH 7.4, 10 mM magnesium chloride (MgCl_2_), 150 mM NaCl, 0.05% Tween 20, in PCR tubes, at room temperature for 10 min. Standard treated capillaries (NanoTemper Technologies) were loaded and the measurements were performed at 22°C, 20% LED power and 40% microscale thermophoresis power, 20 s laser‐on time, 1 s laser‐off time. Owing to the physico‐chemical properties of the LCO, the aforementioned procedure was modified to avoid sticking problems and insertion into micelles. Therefore, a range of LCO concentrations was prepared by a 1 : 1 serial dilution in 50% ethanol. The incubation with the labelled protein (20 nM) was performed in 25 mM sodium cacodylate buffer pH 6.0, 1 mM MgCl_2_, 1 mM calcium chloride, 250 mM sucrose, 0.175% Fos‐choline‐10, 0.5% ethanol (final concentration), in a low‐binding microtitre plate, at room temperature for 10 min. Premium treated capillaries (NanoTemper Technologies) were loaded and the measurements were performed at 22°C, with the settings used for COs except that a 40% LED power was used. All the experiments were repeated at least twice with two independent protein preparations obtained from two bacterial cultures. Binding data were analysed using MO.Affinity Analysis software (NanoTemper Technologies).

### Chitinase protection assay

Either *Fusarium oxysporum *f. sp. *lycopersici* or *Trichoderma viride* were used in this assay. In detail, fungal spores were recovered from PDA plates using 2 ml of sterile distilled water. Spore suspension was adjusted to 10^5^ spores per millilitre and volumes of 40 μl were used to fill in a 96‐well plate, which was incubated overnight at room temperature. After overnight incubation, spore germination was checked by microscopy and treated with 10 µl water, SEC buffer, 50 µM AVR4, 50 µM RiSLM or 100 µM RiSLM and incubated at room temperature for 2 h. After incubation, 10 µl of tomato (*Solanum lycopersicum*) apoplastic fluid preparation was applied into each well and incubated for another 4 h. Integrity of fungal hyphae were observed using a Leica AU5500B microscope (Leica Microsystems, Wetzlar, Germany). Two replicates were used for each treatment.

### RNA isolation and quantitative PCR

For all experiments, total RNAs were isolated using a Qiagen plant RNA mini kit or Omega EZNA Plant RNA kit (Omega Bio‐Tek, Norcross, GA, USA) following the manufacturer’s instructions, including on‐column DNAse treatment. Either 1 µg or 500 ng total RNA was used for reverse transcription using an iScript cDNA synthesis kit (Bio‐Rad). Quantitative PCR (qPCR) was done using an iQ SYBR Green Supermix (Bio‐Rad) on a Bio‐Rad CFX real‐time platform. All plant and fungal genes were normalized using *M. truncatula* or *Rhizophagus irregularis elongation factor 1α*. Milli‐Q water was used as negative control in all qPCR analyses. The primers used are shown in Table S1.

### Defence and symbiotic gene expression analyses

*Medicago truncatula* genotype R108 was used. Seeds were surface sterilized and vernalized at 4°C for 2 d. One‐day‐germinated seedlings were grown in liquid M medium as described by Nars *et al.* (2013) for 5 d. M medium was replaced by Milli‐Q water before all treatments. In order to study whether LysM effector interferes with CO signalling, 100 nM chitotetraose (CO4; Megazyme, Wicklow, Ireland) or chitooctaose (CO8; IsoSep, Tullinge, Sweden) in combination with either 100 nM or 500 nM LysM effector was used. In order to study whether LysM effector interferes with LCO signalling, 10 nM sulphated LCOs (sLCOs) or nonsulfated LCOs (nsLCOs), in both cases C16 : C18 = 1 : 1, in combination with either 10 nM or 50 nM RiSLM was used. All samples were harvested at 1 h after treatment. Three defence marker genes (*MtEPI* (NAD‐dependent epimerase/dehydratase), *MtPAL* (phenylalanine ammonia lyase), and *MtTHA* (thaumatin)), and three symbiotic marker genes (*MtPUB1*, *MtTUBB1*, and *MtVAPYRIN*) were used to determine defence or symbiotic responses, respectively. For all qPCRs, iQ SYBR Green Supermix (Bio‐Rad) and the Bio‐Rad CFX Real‐Time System were used.

### Reactive oxygen species assay

*Medicago truncatula* genotype A17 was used. Sterilized seeds were grown on Faharaeus medium for 5 d. Roots were cut into *c.* 0.5 mm pieces and incubated overnight in 150 µl Milli‐Q water in a black 96‐well polystyrene plate before treatment. Five root pieces were used in each biological replicate. To ROS, Milli‐Q water was replaced by 100 µl Milli‐Q water containing 0.5 mM L‐012 and 10 µg ml^−1^ horseradish peroxidase with 1 µM CO8. To study the role of RiSLM on ROS generation, equal molar (1 µM) RiSLM protein was used. Chemiluminescence was measured using a microplate luminometer (Clariostar, BMG LABTECH GmbH, Ortenberg, Germany) for 0.5 h. At least six biological replicates were used each time, and this experiment was repeated independently for three times with similar results. Similarly, 100 nM flg22 (Genscript, Leiden, the Netherlands) was applied with or without 400 nM RiSLM.

### Host‐induced gene silencing

To generate the silencing construct, 187 bp messenger RNA sequence of *RiSLM* was amplified and cloned into the pDONR221 entry vector. Because the CaMV 35S promoter was previously reported to be less active in ARB cells, we first replaced the 35S promoter from the pK7GWIWG2 (II) RR vector (Limpens *et al.*, 2004) by the AtEF1α promoter (Auriac & Timmers, 2007). Next, the silencing fragment of *RiSLM* was cloned into the modified pK7GWIWG2 (II)‐AtEF1α RR vector using LR clonase II (Invitrogen) to get the final silencing construct. *Medicago* A17 was used for hairy root transformation, and the empty vector was used as control. Hairy root transformations were done according to Limpens *et al.* (2004). After transformation, composite plants were individually transplanted into SC10 RayLeach cone‐tainers (Stuewe & Sons Inc., Tangent, OR, USA) with premixed sand : clay (1 : 2, v/v) mixture. Four hundred spores of *R. irregularis* DAOM197198 (Agronutrition, Parc Activestre, Carbonne, France) were used as inoculum for each cone and placed directly below the seedlings upon planting. Plants were watered using half Hoagland medium with full nitrogen (N), as used for our previous research, and grown in a plant growth chamber at 21°C (16 h : 8 h, light : dark) (Zeng *et al.*, 2018). After 4 wk, transgenic roots were harvested based on the DsRed marker expression. Transgenic roots were chopped in to *c.* 2 cm fragments, mixed thoroughly, and separated into two parts: one for microscopy and the other for RNA isolation.

### RiSLM overexpression

For the overexpression of RiSLM, a construct was made using Golden Gate cloning (Engler *et al.*, 2014). Lotus ubiquitin 1 promotor, MtBCP1 signal peptide, RiSLM CDS without signal peptide, and 35S terminator level 0 modules were combined into the level 1 position F1 acceptor vector pICH47732. A position two AtUB10::DsRed gene was then assembled with the aforementioned LjUB1::BCPsp::RiSLM gene into the level 2 backbone pAGM4723. The level 2 construct was introduced into *Agrobacterium rhizogenes* MSU440 and then transformed into *Medicago* plants by hairy root transformation. A dummy level 1 vector (pICH54011) combined with AtUB10::DsRed into pAGM4723 was used as empty vector control. Plants were harvested after 3 wk inoculation with 400 *R. irregularis* spores, as described earlier.

### Wheat germ agglutinin staining and microscopy

Mycorrhized roots were first cleared in 10% potassium hydroxide at 90°C for 20 min. Roots were then washed twice with phosphate‐buffered saline (150 mM NaCl, 10 mM sodium hydrogen phosphate, and 1.8 mM KH_2_PO_4_, pH 7.4) followed by incubation in 0.2 µg ml^−1^ wheat germ agglutinin (WGA)‐Alexa Fluor^®^ 488 in PBS at 4°C overnight. Microscopy was done using a Leica AU5500B microscope. Mycorrhization was quantified as previously described (Trouvelot *et al.*, 1986).

### Phylogeny

For phylogenetic analyses, amino acid sequences of LysM effectors or LysM domains were aligned using the Mafft plugin in Geneious 8.1.9 (https://www.geneious.com). The phylogenetic tree was generated using tree builder in Geneious 8.1.9 (https://www.geneious.com). For all analyses, the neighbour‐joining tree method was used.

### Yeast signal sequence trap

Full‐length RiSLM was cloned using *Eco*RI‐*Not*I restriction sites into the pYST‐02 vector (Kloppholz *et al.*, 2011). *Saccharomyces cerevisiae* strain Y02321 (Mat a; his3Δ1; leu2Δ0; met15Δ0; ura3Δ0; YIL162w::kanMX4) (Euroscarf, Scientific Research and Development GmbH, Oberursel, Germany) was transformed using the standard lithium acetate/single‐stranded carrier DNA/polyethylene glycol method and selected on SD/Leu plates. Subsequently, transformants were plated on sucrose selection medium (6.7 g l^−1^ yeast N base without amino acids, 1.85 g l^−1^ Drop‐out mix minus leucine, 2% sucrose, 0.025% glucose, 2% agar) and incubated at 30°C for 3 d.

### RNA sequencing analyses

RNA sequencing (RNAseq) data were collected from the following databases: NCBI gene expression omnibus http://www.ncbi.nlm.nih.gov/geo/query/acc.cgi?acc=GSE99655 for data from *Medicago*, chives, and *Nicotiana benthamiana* as well as laser microdissected ARBs and IRM; extraradical mycelium (ERM) and germinating spores from DDBJ database DRA002591; *Lotus japonicus* and tomato from DDBJ database DRA005187. RNAseq data were analysed as described by Zeng *et al.* (2018). Quantification was done using default setting in CLC Genomics Workbench 10.0.1.

### Data availability

Genomic or coding sequences of AM‐secreted LysM (SLM) proteins mentioned in this research can be retrieved in Genbank under the following accession nos.: RiSLM, XM_025315479; RcSLM, KU305772; RiSLM_A1, MN520639; RiSLM‐1_A4, MN520643; RiSLM‐2_A4, MN520642; RiSLM‐3_A4, MN520641; RiSLM‐4_A4, MN520640; RiSLM_C2, MN520636; RiSLM_B3, MN520637; GmSLM, MN520644; GrSLM, MN520645.

## Results

### A *Rhizophagus irregularis* LysM effector highly expressed in intraradical mycelium

**Figure 1:**
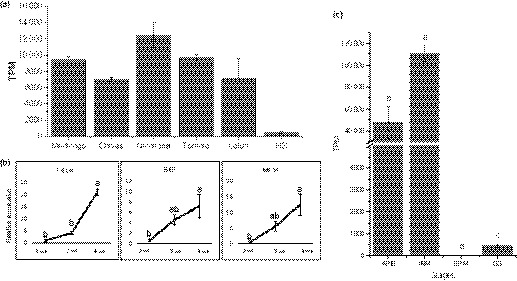
*RiSLM*) is highly induced in intraradical mycelium (IRM) in a wide range of host plants. (a) *RiSLM* is highly induced in five hosts *Medicago truncatula* (Medicago), *Allium schoenoprasum* (Chives), *Nicotiana benthamiana* (Nicotiana), *Solanum lycopersicum* (Tomato) and *Lotus japonicus* (Lotus) compared with germinating spores (GS). Transcripts per million (TPM) was used to represent expression levels. Error bars represent SE from either three replicates (for Medicago, Chives, and Nicotiana) or two replicates (for Lotus and Tomato), collected from independent RNA sequencing (RNAseq) experiments. (b) Quantitative PCR analysis, showing that *RiSLM* (relative to *Rhizophagus irregularis elongation factor 1α*, *RiEF*) is induced during arbuscular mychorrizal colonization and arbuscule (ARB) development as represented by relative expression of *RiEF* and the ARB‐specific phosphate transporter (*MtPT4*) normalized by *Medicago* *elongation factor 1α* (*MtEF*). Error bars represent SE from three biological replicates. (c) *RiSLM* expression based on RNAseq analyses of laser microdissected ARBs and IRM compared with extraradical mycelium (ERM) and GS. Error bars represent SE from three biological replicates. For all figures, different characters indicate significant differences (least significant difference *P* < 0.05)

Fungal pathogens use LysM effectors to counteract chitin‐based immune responses to colonize plants (Sánchez‐Vallet *et al.*, 2015). By analysing the predicted secretome of the model AM fungus *R. irregularis* DAOM197198, a small secreted effector, consisting only of a single LysM domain, was identified and named RiSLM (Tisserant *et al.*, 2013; Sędzielewska Toro & Brachmann, 2016; Zeng *et al.*, 2018; Schmitz *et al.*, 2019). Our transcriptome analysis showed that this effector is one of the most highly expressed fungal genes in the symbiosis, with at least three evolutionarily distantly related plant species: *Medicago*, *Nicotiana benthamiana*, and *Allium schoenoprasum* (Zeng *et al.*, 2018). We additionally mined publicly available transcriptome data of *R. irregularis* and found that *RiSLM* is also highly expressed in the symbiosis with tomato and *L. japonicus* (Sugimura & Saito, 2017) (Fig. 1a). The high expression level of *RiSLM* in such a wide range of host species suggests that RiSLM plays a fundamental role during the symbiosis. A time‐course experiment in *Medicago* revealed that *RiSLM* expression correlates with fungal colonization and abundance, as reflected by the expression of *R. irregularis Elongation Factor 1α* (*RiEF*) and the ARB‐specific phosphate transporter (*MtPT4*; Javot *et al.*, 2007) (Fig. 1b). Stage‐specific transcriptome analyses in *Medicago* further indicated that *RiSLM* is the highest expressed effector gene in IRM (Fig. 1c). Expression of *RiSLM* was also induced in germinating spores upon treatment with the strigolactone analogue GR24 and rice root exudates (Tsuzuki *et al.*, 2016; Nadal *et al.*, 2017), suggesting that RiSLM may play a role already upon fungal contact at the epidermis. To verify that RiSLM can be secreted by the fungus, we used a yeast signal sequence trap system, in analogy to Kloppholz *et al.* (2011). Expressing the full‐length RiSLM protein including its endogenous signal peptide sequence fused to an invertase allowed the mutant yeast strain Y02321, which cannot secrete invertase by itself, to efficiently grow on sucrose‐containing medium (Fig. S1). This indicates that RiSLM is indeed a secreted protein.

### LysM effector genes are a conserved feature in arbuscular mycorrhizal fungi

**Figure 2:**
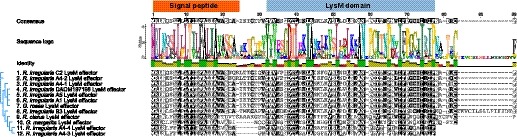
Lysin motif (LysM) effectors are a conserved feature of arbuscular mychorrizal (AM) fungi. LysM effectors from different AM fungal species/isolates, including six *Rhizophagus irregularis* isolates (DAOM197198/RiSLM, A1, A4, A5, B3, and C2), *Rhizophagus clarus*, *Gigaspora rosea*, and *Gigaspora margarita*, were aligned using Mafft and grouped using Geneious tree builder (https://www.geneious.com). Signal peptide and LysM domain are marked.

To determine whether the presence of a LysM effector gene is a conserved feature of AM fungi, we searched for RiSLM homologues in the publicly available genome or transcriptome sequences of five additional *R. irregularis* isolates (Ropars *et al.*, 2016; Chen *et al.*, 2018) and of the different AM species *Rhizophagus clarus* (Sędzielewska Toro and Brachmann, 2016), *Gigaspora margarita* and *Gigaspora rosea* (Tang *et al.*, 2016; Salvioli *et al.*, 2016). In all AM fungal genomes/transcriptomes we could detect the presence of LysM effectors, containing a signal peptide followed by a single LysM domain (Table S2). Most AM fungal species/isolates harbour only one LysM effector. However, we detected four LysM effector‐coding genes in the *R. irregularis* A4 genome (Fig. 2). We noticed that there is significant variation at the RiSLM amino acid level in the different *R. irregularis* isolates, in agreement with the analyses by Schmitz *et al.* (2019). Strikingly, even higher divergence is observed among RiSLM protein sequences of different *R. irregularis* isolates than between the LysM effector from *R. irregularis* and *G. rosea* (Fig. 2), which leads us to speculate that there may be significant levels of selection or adaptation to different plant hosts (e.g. to avoid subsequent effector‐triggered immune responses). Nevertheless, these analyses indicate that the presence of a LysM effector gene is a conserved feature in AM fungi.

Comparison of AM fungal LysM domains with pathogenic fungal LysM domains showed that most AM LysM effectors contain two conserved cysteine residues in the LysM domain (Fig. S2), in contrast to only one or no cysteines in the LysM domains of pathogenic LysM effectors. These cysteine residues may be crucial to form disulphide bridges to stabilize the protein or to form protein complexes. In addition, significant enrichment of positively charged lysine and arginine residues was observed in most AM LysM domains (Fig. S2), suggesting that ionic interactions may be important for proper function of AM LysM effectors. Alternatively, positive charged residues in the LysM domain may be required to recruit the effectors to the negatively charged host membrane to interact with potential host receptors.

### RiSLM binds chitin and chitooligosaccharides

**Figure 3:**
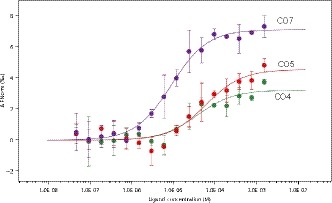
RiSLM) binds chitin in the micromolar range. Chitotetraose (CO4), chitopentaose (CO5), and chitoheptaose (CO7) were used to test binding affinity between RiSLM and chitin‐oligosaccharides using microscale thermophoresis. This revealed affinities of RiSLM for CO4, CO5, and CO7. The *x*‐axis indicates ligand concentration, and the *y*‐axis indicates the change in normalized fluorescence (ΔFnorm (‰)) after binding with different ligands. Error bars represent SD from two independent measurements using two batches of independently purified proteins.

To investigate the function of RiSLM we produced and purified the RiSLM protein, using the Champion pET‐SUMO expression system, from *E. coli* ORIGAMI cells. After affinity purification and size‐exclusion chromatography, we noticed two faint additional bands (of *c.* 14 and *c.* 21 kDa) next to the expected *c.* 7 kDa RiSLM band on a Coomassie‐blue‐stained protein gel (Fig. S3a). These bands may reflect oligomers of RiSLM, as was previously observed for the Slp1 LysM effector from *M. oryzae* and *Vd2LysM* from *V. dahliae* (Mentlak *et al.*, 2012; Kombrink *et al.*, 2017). We first tested the ability of the purified RiSLM protein to bind chitin using affinity precipitation assays with the insoluble carbohydrates chitin, chitosan, xylan, and cellulose. This showed that the majority of the RiSLM protein was precipitated with insoluble chitin, but not (or hardly) with cellulose or xylan (Fig. S3b). Incubation with insoluble chitosan (deacetylated chitin) also resulted in precipitation of RiSLM, although not as strong as with chitin. This indicates that RiSLM has the highest affinity for acetylated chitin residues. To better study the affinity of RiSLM for COs of different lengths we performed microscale thermophoresis experiments. This revealed a binding affinity *K*_d_ of 8.8 ± 1.2 µM for CO7, and slightly lower for CO4 (37.1 ± 1.4 µM) and CO5 (59.4 ± 2.1 µM) (Fig. 3). No binding was observed for their deacetylated counterpart (Fig. S4). These analyses show that RiSLM can bind COs of various lengths, of which especially longer chain Cos, such as CO7 or CO8, are known to be potent elicitors of chitin‐triggered immunity in plants.

### RiSLM can protect fungal hyphae against degradation by plant chitinases

**Figure 4:**
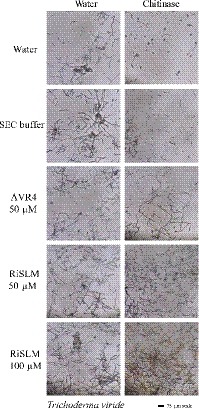
RiSLM) protects hyphae from the pathogenic fungus *Trichoderma viride* against plant chitinases. Germinated hyphae are treated with 50 µM AVR4, 50 µM RiSLM, or 100 µM RiSLM and incubated at room temperature for 2 h and subsequently treated with a tomato chitinase preparation or water for 4 h. Water or buffer used for size‐exclusion chromatography (SEC) were used as negative controls. The Avr4 effector from *Cladosporium fulvum* was used as positive control.

Fungal LysM effectors from pathogens have been found to either protect fungal cell walls from apoplastic chitinases or to suppress chitin‐triggered immunity, with some LysM effectors possessing both activities to support fungal infection (de Jonge *et al.*, 2010; Marshall *et al.*, 2011; Mentlak *et al.*, 2012; Lee *et al.*, 2014; Kombrink *et al.*, 2017). To test whether RiSLM has the ability to protect against plant chitinases, we tested the effect of RiSLM on hyphal integrity of *T. viride* and *F. oxysporum* f. sp. *lycopersici* upon treatment with tomato chitinases. Germinated *T. viride* and *F. oxysporum* f. sp. *lycopersici* spores were incubated for 4 h with a chitinase extract from tomato leaves in the presence or absence of 50 or 100 µM RiSLM. As a positive control, we used the chitin‐binding AVR4 effector from *C. fulvum*, which has been shown to protect fungal hyphae in a similar assay (van den Burg *et al.*, 2006). As can be seen from Figs 4 and S5, fungal hyphae were still intact after treatment with the chitinase extract in the presence of both CfAVR4 and RiSLM, whereas control treatments resulted in their degradation, showing that RiSLM can protect fungal hyphae against the hydrolytic activity of plant chitinases.

### RiSLM suppresses chitin‐triggered immune responses

**Figure 5:**
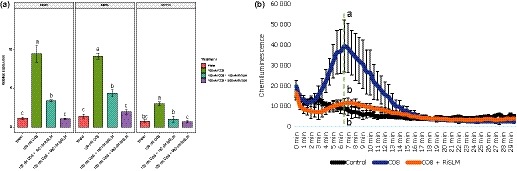
RiSLM) suppresses chitooctaose (CO8)‐triggered plant responses. (a) RiSLM suppresses CO8‐triggered defence marker gene induction in *Medicago truncatula* R108 roots treated with 100 nM CO8 in combination with either 100 or 500 nM RiSLM for 1 h. Three defence marker genes, EPI, PAL, and THA, were used to monitor defence responses by quantitative PCR. Error bars represent SE from three biological replicates. (b) RiSLM suppresses chitin‐triggered reactive oxygen species burst. *Medicago truncatula* A17 root pieces were treated with 1 µM CO8 with or without equal molar amounts of RiSLM. Error bars represent SE from six6 biological replicates. For both figures, different letters indicate significant difference (least significant difference *P* < 0.05) between different treatments.

Next, we tested whether RiSLM can also suppress chitin‐triggered immune responses in the plant. As a first assay, we studied the ability of RiSLM to suppress CO8‐induced defence gene induction in *Medicago* roots. In this assay (Nars *et al.*, 2013), freshly germinated *Medicago* seedlings were incubated in liquid M medium for 5 d at 21°C, after which the plants were treated with 100 nM CO8 in the presence or absence of either equimolar (100 nM) or 5× excess (500 nM) of RiSLM protein. Roots were harvested 1 h after treatment, and the expression of three defence marker genes, *MtEPI* (NAD‐dependent epimerase/dehydratase), *MtPAL* (phenylalanine ammonia lyase) and *MtTHA* (thaumatin), was determined by real‐time PCR analysis. Upon 1 h CO8 treatment, all marker genes were markedly induced. This induction was suppressed by 100 nM RiSLM, with 500 nM RiSLM showing an even stronger inhibitory effect, indicating that RiSLM suppresses CO8‐induced defence gene expression in a dose‐dependent manner (Fig. 5a).

As a second assay, we measured the ability of RiSLM to inhibit CO8‐induced ROS production using a chemiluminescence assay. In the absence of RiSLM, 1 µM CO8 induced a clear ROS burst in 0.5–1 cm *Medicago* root sections (Fig. 5b). However, the ROS burst was greatly reduced upon co‐treatment with equimolar (1 µM) amounts of RiSLM, showing that RiSLM is able to suppress chitin‐triggered ROS production (Fig. 5b). To rule out that RiSLM may have a general inhibitory effect on pathogen‐associated molecular‐pattern‐triggered defence responses, we tested whether RiSLM would inhibit flg22‐induced ROS production. No inhibition of an ROS burst induced by 100 nM flg22 was observed in *Medicago* roots when 4× excess RiSLM was used (Fig. S6). Taken together, these results indicate that RiSLM can suppress chitin‐triggered immune responses.

### RiSLM does not significantly block symbiotic signalling

**Figure 6:**
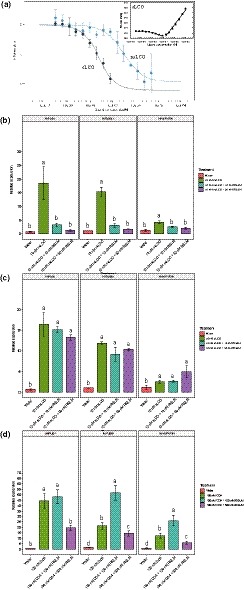
RiSLM) does not block symbiotic responses. (a) Binding of RiSLM to lipo‐chitooligosaccharides (LCOs) LCO‐IV(C18:1, sulphated LCO (sLCO)) and LCO‐IV(C18:1, nonsulphated LCO (nsLCO)) (black and blue circles, respectively) as determined by microscale thermophoresis and corresponding to the same binding event. Inset: two binding events occurring at low (0.1–100 nM) and high concentrations (100 nM to 10 µM without reaching saturation) of LCO‐IV(C18:1, S). Error bars represent SD from two independent measurements using two batches of independently purified proteins. (b, c) Quantitative PCR (qPCR) analysis of *Medicago truncatula* R108 roots treated with (b) 10 nM nonsulphated myc‐LCOs or (c) sulphated myc‐LCOs in combination with either 10 or 50 nM RiSLM. Three symbiotic marker genes, *PUB1*, *TUBB1*, or *Vapyrin*, were used to monitor symbiotic responses induced by LCOs, using Med*ic*ago *elongation factor 1α* (*MtEF*) as reference gene. Error bars represent SE from three biological replicates. (d) qPCR analysis of *Medicago* R108 roots treated with water (*n* = 3) or 100 nM chitotetraose (CO4, *n* = 3) in combination with either 100 nM (*n* = 4) or 500 nM RiSLM (*n* = 3), showing that RiSLM does not strongly suppress CO4‐triggered symbiotic marker gene induction. Different letters indicate significant difference (least significant difference *P* < 0.05) between different treatments.

Besides chitin oligomers that trigger immune responses, AM fungi also produce chitin‐derived LCOs (Myc‐LCOs) and short‐chain COs (CO4/5), collectively called Myc factors, that activate the common symbiotic signalling pathway to establish the symbiosis (Maillet *et al.*, 2011; Genre *et al.*, 2013). Our study already indicated that RiSLM can bind CO4 and CO5 in the micromolar range (Fig. 3), raising the question whether RiSLM might interfere with the signalling role of Myc factors. To investigate this, we first additionally tested the affinity of RiSLM for Myc‐LCOs. *Rhizophagus irregularis* produces both sulphated and nonsulphated tetrameric or pentameric LCOs with variable length/saturation of the fatty acyl chain (Maillet *et al.*, 2011). Microscale thermophoresis analyses of RiSLM with either sulphated or nonsulphated tetrameric (C18:1) LCOs showed that RiSLM binds these Myc‐LCOs. LCO‐IV(C18:1, S) binding curve analysis revealed two binding events (inset Fig. 6a). The first one occurs at concentrations ranging from 0.1 to 100 nM, and the other one is observed at higher LCO concentrations, with a much larger fluorescence signal amplitude but without reaching saturation. LCO‐IV(C18:1) binding to RiSLM resulted in a single binding event exhibiting a feature similar to the first event of LCO‐IV(C18:1, S) binding. Therefore, by referring to the same binding event, it can be deduced from the plots reported in Fig6a (main panel) that LCO‐IV(C18:1, S) exhibits a higher affinity (*K*_d_ ~ 15 nM) than LCO‐IV(C18:1) (*K*_d_ ~ 240 nM). The second binding event observed for LCO‐IV(C18:1, S) could be due to multimerization; and because it occurs at high concentrations, it is probably not biologically relevant.

Next, we tested whether co‐application of RiSLM can interfere with symbiotic marker gene induction by the different Myc factors. Therefore, we used the same experimental set‐up as before for monitoring the defence gene induction by CO8, and roots were harvested 1 h after Myc factor application. For Myc‐LCO application, equal molar amounts of C16:0 and C18:1 sLCO were mixed and used as Myc‐sLCO (10 nM) preparation and the same was done for C16:0 and C18:1 nsLCOs (Myc‐nsLCOs). For CO4 we used 100 nM, and in all cases RiSLM was applied in equimolar amounts as well as in a 5× excess. To quantify symbiotic responses, three marker genes (MtPUB1, MtTUBB1, and MtVAPYRIN) were selected that have previously been shown to be induced by Myc‐LCOs (Czaja *et al.*, 2012; Camps *et al.*, 2015). *MtPUB1* encodes an LCO‐induced ubiquitin E3 ligase, which interacts with LYK3 and DMI2 receptor kinases to limit rhizobial and AM fungal infection (Vernié *et al.*, 2016). *VAPYRIN* is thought to be involved in the regulation of exocytosis required for colonization and widely used as a symbiotic marker (Pumplin *et al.*, 2010; Murray *et al.*, 2011). The Myc‐LCOs induced *MtTUBB1* encodes a putative β‐tubulin (Czaja *et al.*, 2012; Camps *et al.*, 2015). Our results indicated that RiSLM already strongly inhibited nsLCO‐triggered symbiotic gene induction when applied at equimolar amounts (Fig. 6b). However, RiSLM did not show any inhibition of sLCO‐triggered symbiotic responses, even when applied at five times higher levels (Fig. 6c). For CO4 we observed a *c.* 50% reduced induction for *MtPUB1* and *MtVAPYRIN* and *c.* 30% reduced induction for *MtTUBB1*, when applied in excess (Fig. 6d). However, co‐application of RiSLM with equimolar amounts of CO4 strikingly enhanced the expression of *MtTUBB1* and *MtVAPYRIN* by a factor of 2.5.

Taken together, our data indicate that RiSLM can suppress and help evade chitin‐triggered immune responses while still allowing sufficient symbiotic signalling to colonize the roots.

### RiSLM plays a positive role in root colonization

To determine whether RiSLM indeed has a positive role during root colonization, we attempted to overexpress RiSLM or to knock down its expression via host‐induced gene silencing (HIGS). Although AM fungi currently cannot be stably transformed due to their coenocytic nature, HIGS has previously been successfully used to study the function of several fungal genes (Helber *et al.*, 2011; Tsuzuki *et al.*, 2016; Kikuchi *et al.*, 2016).

To determine whether overexpression of *RiSLM* could increase colonization, we overexpressed RiSLM in *Medicago* roots under control of the AtUBIQUITIN3 promoter. We noticed that expression of the full‐length predicted *R. irregularis* effectors containing their endogenous signal peptides *in planta* typically leads to an accumulation in the endoplasmic reticulum (data not shown). Therefore, to efficiently direct the effector to the apoplast, we fused the mature RiSLM protein to the signal peptide of MtBCP1 (Ivanov & Harrison, 2014). We confirmed the overexpression of RiSLM by real‐time PCR (Fig. S7b). Three weeks after inoculation there were no significant differences observed in either fungal abundance or ARB abundance compared with empty vector control roots (Fig. S7a,c). This likely suggests that the endogenous levels of *RiSLM* expression are sufficient to support successful infection, although we did not verify the overexpression at the protein level.

**Figure 7:**
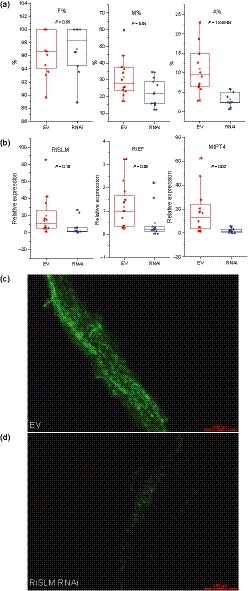
Host‐induced gene silencing of *RiSLM*) reduces mycorrhization in *Medicago truncatula*. (a) Frequency (F%) remains the same, whereas mycorrhization intensity in the root (M%) and arbuscule abundance in the root (A%) are reduced in *RiSLM*‐silenced roots. For the empty vector control (EV), 12 biological replicates were used. For *RiSLM* RNA interference (RNAi), 10 biological replicates were used. (b) Quantitative PCR analysis of control and RNAi roots showing *RiSLM* expression level relative to *Rhizophagus irregularis elongation factor 1α* (*RiEF*) and *MtPT4* expression levels relative to Medicago *elongation factor 1α* (*MtEF*). Successful silencing of *RiSLM* (relative to *RiEF*) reduces *RiEF* and *MtPT4* expression (relative to *MtEF*), indicating reduced mycorrhization and arbuscule abundance. Twelve replicates (individual transgenic roots) were used for EV. Eleven replicates were used for RNAi. Student’s *t*‐test *P*‐values are indicated for (a) and (b). For all box plots, boxes represent interquartile range (IQR) and whiskers represent 1.5IQR. (c, d) Wheat germ agglutinin‐Alexa Fluor 488 staining of mycorrhization in (d) *RiSLM*‐silenced roots and (c) control roots (c). Bars, 200 µm.

To trigger HIGS, we introduced a hairpin construct targeting *RiSLM* driven by the constitutive *At*EF1α promoter (Auriac & Timmers, 2007) into *Medicago* roots via *Agrobacterium rhizogenes*‐mediated transformation. An empty vector was used as control. Transgenic roots were selected based on the co‐expression of a *DsRED1* marker gene. The level of AM colonization was scored 4 wk after inoculation with *R. irregularis* spores (Trouvelot *et al.*, 1986) in transgenic roots stained with WGA‐Alexa Fluor 488. This showed that roots carrying the silencing construct had significantly lower fungal colonization and ARB levels than the empty vector control roots (Fig. 7a,c,d). This correlated with reduced expression of *RiEF*, a marker for total fungal abundance, and expression of the ARB‐specific phosphate transporter *MtPT4* used as marker for ARB abundance (Javot *et al.*, 2007) (Fig. 7b). Consistently, *RiSLM* expression was also significantly reduced in the HIGS roots compared with empty vector control roots (Fig. 7b). This indicates that RiSLM plays a positive role in AM fungal root colonization.

## Discussion

Here, we show that the AM fungus *R. irregularis* secretes the chitin‐binding LysM effector RiSLM to facilitate efficient intraradical colonization. A secreted single LysM domain containing effector is present in a wide range of AM species, suggesting that the involvement of a LysM effector is a conserved feature in AM fungi. The variation in protein sequence of these LysM effectors in different isolates suggests a potential diversifying selection that has also been reported for LysM effectors from pathogenic fungi (Marshall *et al.*, 2011; Schmitz *et al.*, 2019). Purified RiSLM was able to protect fungal hyphae from hydrolysis by plant chitinases and to suppress chitin‐triggered immune responses in *Medicago* roots. A combination of both activities has also been reported for the LysM effectors Mg3LysM from *Z. tritici* and Vd2LysM from *V. dahliae*, which contribute to the virulence of these pathogenic fungi (Marshall *et al.*, 2011; Kombrink *et al.*, 2017). This indicates that LysM effectors are used by both symbiotic and pathogenic fungi to colonize plants (Kombrink & Thomma, 2013).

RiSLM was able to bind both chitin and COs as well as to polymeric chitosan, a deacetylated form of chitin, in agreement with Schmitz *et al.* (2019). Chitin is generally considered to be a more potent trigger of immune responses than chitosan in many plants, and several pathogenic fungi deacetylate their chitin as a strategy to evade the immune system (El Gueddari *et al.*, 2002; Rovenich *et al.*, 2016). However, chitosan has also been reported to be a strong inducer of immunity in some plants (Sánchez‐Vallet *et al.*, 2015). We noticed previously that many chitin deacetylases that are expressed in ERM are downregulated when the fungus grows intraradically in *Medicago* (Zeng *et al.*, 2018). Therefore, acetylated chitin is likely the most dominant form when *R. irregularis* grows inside the root and where *RiSLM* is most strongly expressed. RiSLM did not bind deacetylated CO4 and CO5 in microscale thermophoresis, suggesting that RiSLM may bind to long‐chain chitosan but not to short‐chain deacetylated COs. Several chitinases and chitosanases have been shown to be induced in roots during AM symbiosis (Pozo *et al.*, 1998). Therefore, the protective role of RiSLM to counteract the activity of chitinases may be important to support hyphal growth inside the root. Whether RiSLM forms a protective layer around the fungal cell wall or directly impairs the activity of plant chitinases remains to be demonstrated. This is currently hampered by the fact that biological relevant concentrations of chitinases and their composition in *Medicago* roots during symbiosis are unknown.

In *Medicago*, chitin‐triggered immune responses require perception by the LysM receptor kinases MtLYK9 and MtLYR4, which are thought to form a receptor complex in analogy to the AtCERK1‐AtLYK5/4 chitin receptor complex in Arabidopsis (Cao *et al.*, 2014; Bozsoki *et al.*, 2017). The orthologue of MtLYK9 in *L. japonicus* is LjLYS6. Affinity studies showed that the LjLYS6 ectodomain binds CO6‐8, with a *K*_d_ of 26 µM for CO7 (Bozsoki *et al.*, 2017). This is only slightly lower than the affinity of RiSLM for CO7, which showed a *K*_d_ of *c.* 9 µM. This affinity is significantly lower than the picomolar affinity of the *C. fulvum* LysM effector Ecp6, purified from *Pichia pastoris*, which contains three LysM domains (de Jonge *et al.*, 2010). Ecp6 was shown to outcompete chitin binding to host receptors in tomato (Sánchez‐Vallet *et al.*, 2013). The relatively low affinity of RiSLM for COs raises the question as to how RiSLM can efficiently interfere with chitin‐triggered immune responses. Depending on the relative concentration of the effector and receptors, it may either sequester COs or, possibly, RiSLM interferes with chitin‐induced receptor dimerization/complex formation. However, it should be noted that the *in vitro* affinity of LysM proteins purified from various heterologous systems can be tricky to compare and may also differ from affinity estimated from biological responses to different ligands. Additional modifications (such as glycosylation) and complex formation with co‐receptors may also increase the *in planta* affinity of the receptors (Chen *et al.*, 2014).

RiSLM was further able to bind a sulphated Myc‐LCO as well as CO4; however, induction of symbiotic gene expression was not strongly inhibited by the co‐application of RiSLM. This is likely due to the higher affinity of the plant receptors for these molecules, although the identity of the receptors in relation to mycorrhization is still elusive. Sulphated Myc‐LCOs are structurally very similar to the Nod factors produced by *Sinorhizobium meliloti*, the rhizobial symbiont of *Medicago*. *Sinorhizobium meliloti* Nod factors contain a sulphate group at the terminal reducing sugar are biologically active at picomolar concentrations and are perceived by the LysM receptors MtNFP and MtLYK3 (Dénarié *et al.*, 1996; Arrighi *et al.*, 2006). An *S. meliloti nodH* mutant, which makes nonsulphated Nod factors, is unable to establish a symbiotic interaction, indicating that *Medicago* has a very high affinity perception system for sLCOs (Roche *et al.*, 1991). This high affinity for sLCO likely explains why *RiSLM*, despite a higher affinity for sLCO‐IV(C18:1, S) (*K*_d_ ~ 15 nM) than for LCO‐IV(C18:1) (*K*_d_ ~ 240 nM), did not interfere with Myc‐sLCOs induced gene expression. Surprisingly, adding equimolar amounts of RiSLM to CO4 appeared to enhance symbiotic signalling (Fig. 5c), suggesting that RiSLM may have an additional role in symbiotic signalling (e.g. interacting with Myc factor receptors). However, we cannot fully rule out the possibility that RiSLM enhances symbiotic signalling by binding long‐chain CO impurities in the CO4 preparate.

In conclusion, we revealed an important role for the secreted LysM effector of *R. irregularis* to suppress or evade chitin‐based immune responses to allow successful colonization of plant roots. RiSLM binding to chitin‐like molecules appears to compete with plant LysM receptors for these molecules to favour more symbiotic responses. Intriguingly, we found that *Medicago* also strongly induces the expression of three secreted single LysM domain proteins (Medtr4g091000, Medtr4g091010, and Medtr4g091020), especially in ARB‐containing cells, which seem to be conserved in other AM hosts (Gaude *et al.*, 2012; Hogekamp & Küster, 2013; Zeng *et al.*, 2018). It will be interesting, therefore, to investigate whether these putative plant LysM effectors play similar or divergent roles in the intracellular colonization of root inner cortex cells by AM fungi.

## Author contributions

TZ, LR‐M, AM and EL conceived and designed experiments. TZ, LR‐M, AM, PW, WvdB, VG, J‐JB and EL performed experiments and/or data analyses. SF and SC provided LCOs. TZ, LR‐M, BPHJT, J‐JB, TB and EL wrote the manuscript. LR‐M and AM contributed equally to this work.

## Supporting information

Please note: Wiley Blackwell are not responsible for the content or functionality of any Supporting Information supplied by the authors. Any queries (other than missing material) should be directed to the *New Phytologist* Central Office.

**Fig. S1 **Yeast signal sequence trap.
**Fig. S2 **Comparison of LysM domains from AM fungal or pathogenic LysM effectors.
**Fig. S3 **Purified RiSLM protein binds chitin and chitosan.
**Fig. S4 **RiSLM does not bind deactylated chitotetraose (CO4) and chitopentaose (CO5) as revealed by microscale thermophoresis.
**Fig. S5 **RiSLM protects hyphae from the pathogenic fungus *Fusarium oxysporum f.sp lycopersici *against plant chitinases.
**Fig. S6 **RiSLM does not suppress flg22‐induced reactive oxygen species (ROS) production.
**Fig. S7** *RiSLM *overexpression does not enhance mycorrhization in *Medicago truncatula*.
**Table S1 **Primers used in this research.
**Table S2 **Coding sequences of LysM effectors from different AM fungal species or isolates.
