# Supplementary material for: A lysin motif effector subverts chitin‐triggered immunity to facilitate arbuscular mycorrhizal symbiosis
Source: New Phytol. 2019 Nov 23;225(1):448–60. doi: 10.1111/nph.16245 (PMC6916333; doi:10.1111/nph.16245)
Supplement: Supplementary file 1 — Fig. S1 Yeast signal sequence trap. Fig. S2 Comparison of LysM domains from AM fungal or pathogenic LysM effectors. Fig. S3 Purified RiSLM protein binds chitin and chitosan. Fig. S4 RiSLM does not bind deactylated chitotetraose (CO4) and chitopentaose (CO5) as revealed by microscale thermophoresis. Fig. S5 RiSLM protects hyphae from the pathogenic fungus Fusarium oxysporum f.sp lycopersici against plant chitinases. Fig. S6 RiSLM does not suppress flg22‐induced reactive oxygen species (ROS) production. Fig. S7 RiSLM overexpression does not enhance mycorrhization in Medicago truncatula. Table S1 Primers used in this research. Table S2 Coding sequences of LysM effectors from different AM fungal species or isolates. [file NPH-225-448-s001.pdf]

***New Phytologist* Supporting Information**

Article title: **A LysM effector subverts chitin-triggered immunity to facilitate arbuscular mycorrhizal symbiosis**

Authors: Tian Zeng, Luis Rodriguez-Moreno, Artem Mansurkhodzaev, Peng Wang, Willy van den Berg, Virginie Gasciolli, Sylvain Cottaz,Sébastien Fort, Bart P.H.J. Thomma, Jean-Jacques Bono, Ton Bisseling, Erik Limpens

Article acceptance date: 24 September 2019

The following Supporting Information is available for this article:

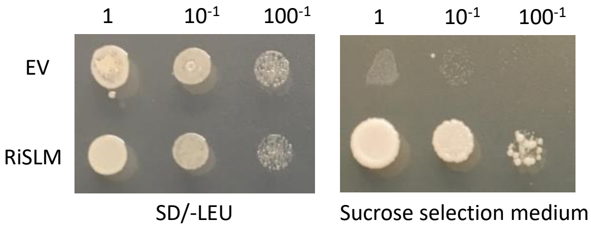

Fig. S1 Yeast signal sequence trap. pYST-02-RiSLM, representing a fusion of full length RiSLM (including its endogenous signal sequence) with an invertase, and the empty pYST-02 vector (EV) were transformed into *Saccharomyces cerevisiae* Y02321, and grown on SD/-Leu (containing glucose) and sucrose selection medium at different dilutions for 3 days at 30 ^{o}C.

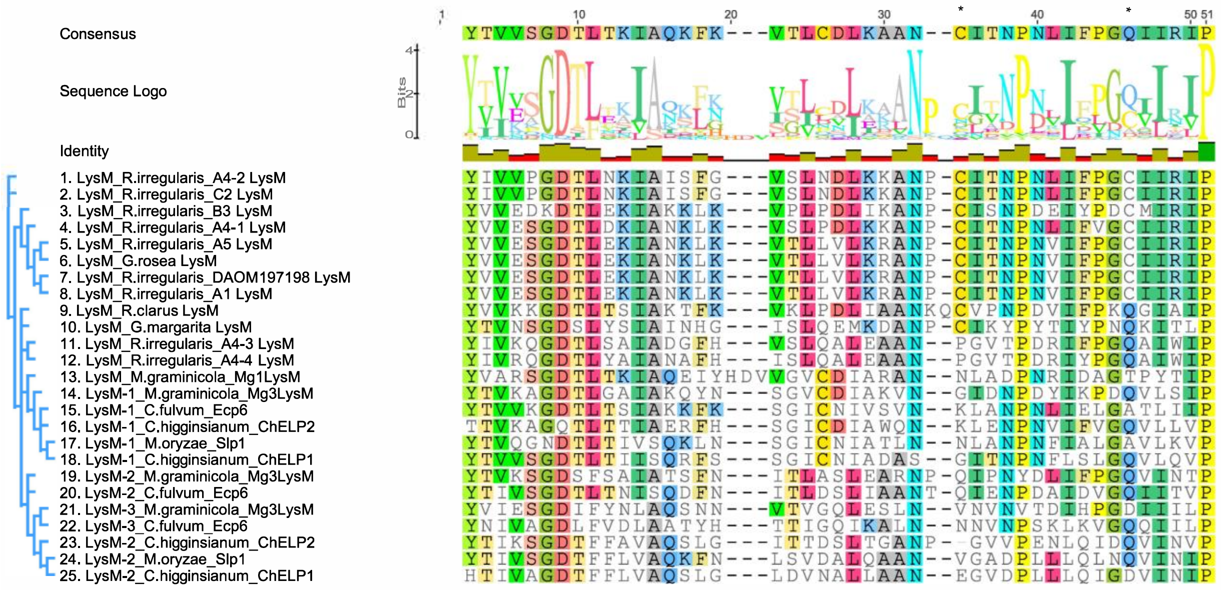

Fig. S2 Comparison of LysM domains from AM fungal or pathogenic LysM effectors.

MAFFT was used to align all amino acid sequences. Neighbour-joining Tree was built using Geneious tree builder.

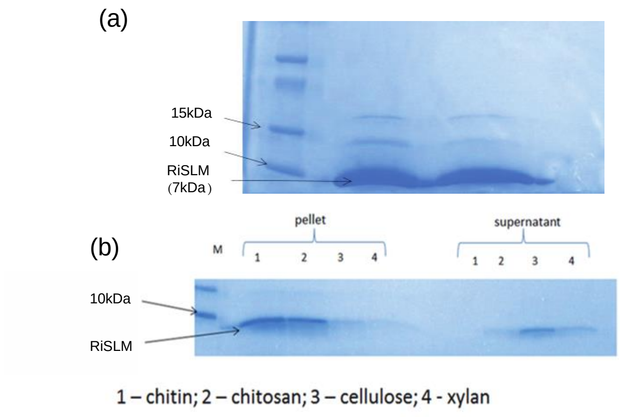

Fig. S3 Purified RiSLM protein binds chitin and chitosan. (a) Coomassie stained protein gel of RiSLM (size ~7kDa) purified from *Escherichia coli* ORIGAMI. (b) Coomassie stained protein gel of affinity precipitation assays showing that RiSLM binds to insoluble chitin and chitosan but not xylan and cellulose. For the latter two polymers the vast majority of the protein is retained in the supernatant.

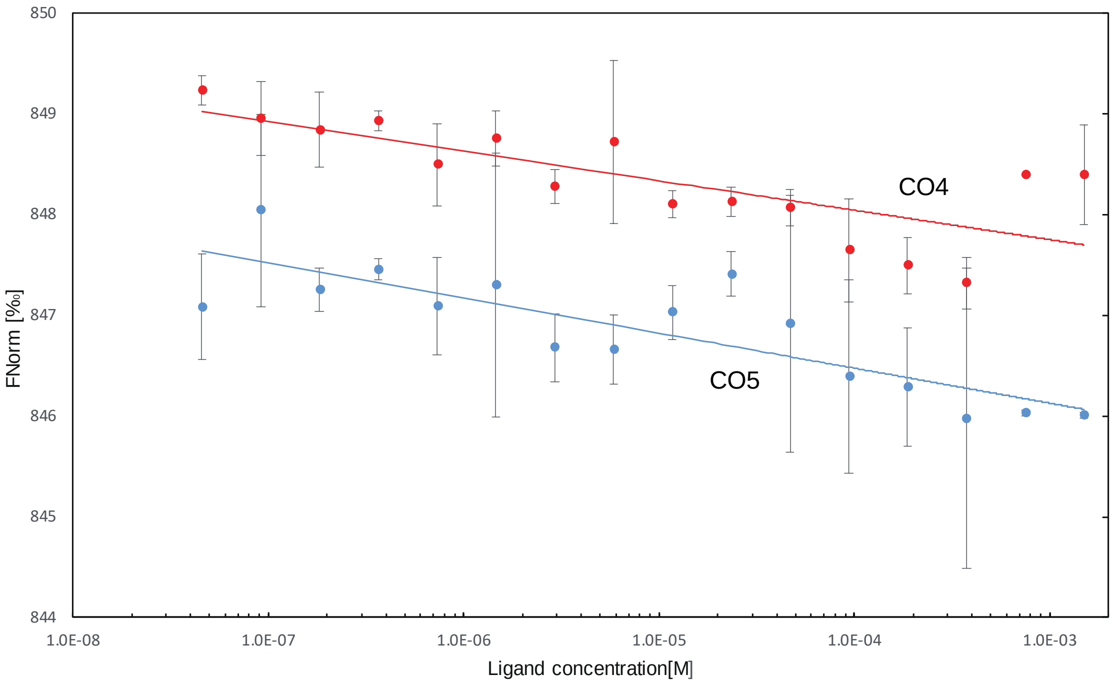

Fig. S4 RiSLM does not bind deactylated chitotetraose (CO4) and chitopentaose (CO5) as revealed by microscale thermophoresis. Binding experiments using microscale thermophoresis do not show any interaction with RiSLM in the range of concentrations tested (up to 1 mM). Error bars represent standard deviation from two independent measurements using two batches of independently purified proteins.

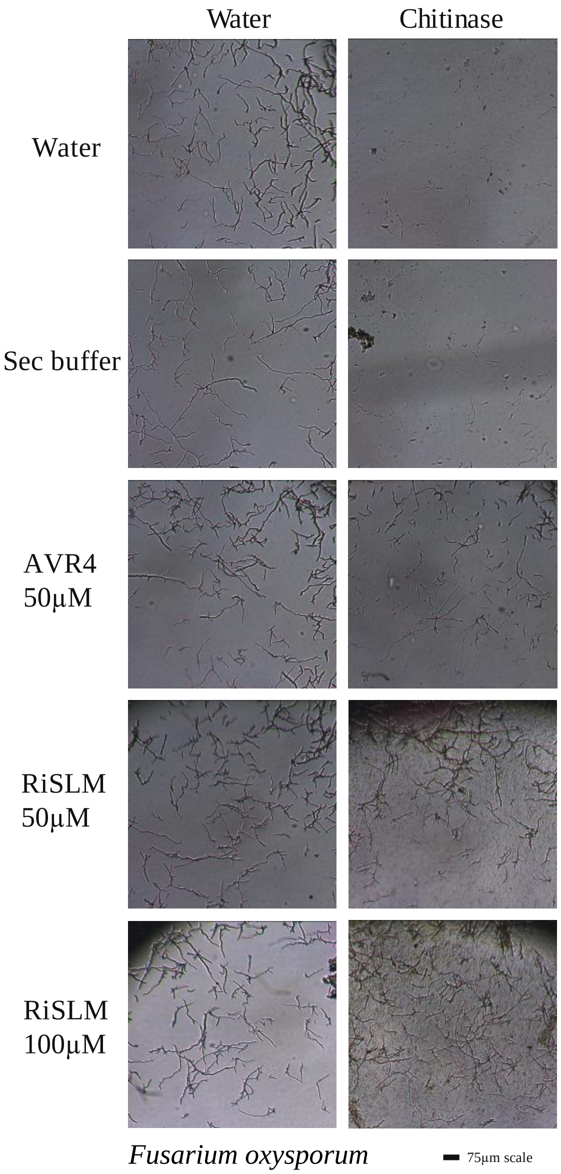

Fig. S5 RiSLM protects hyphae from the pathogenic fungus *Fusarium oxysporum f.sp lycopersici* against plant chitinases. Germinated hyphae are treated with 50 µM AVR4, 50 µM RiSLM or 100 µM RiSLM and incubated at room temperature for 2 hours, and subsequently treated with a tomato chitinase preparation or water for 4 hours. Water or buffer used for size-exclusion chromatography (SEC) were used as negative controls. The Avr4 effector from *Cladosporium fulvum* was used as positive control.

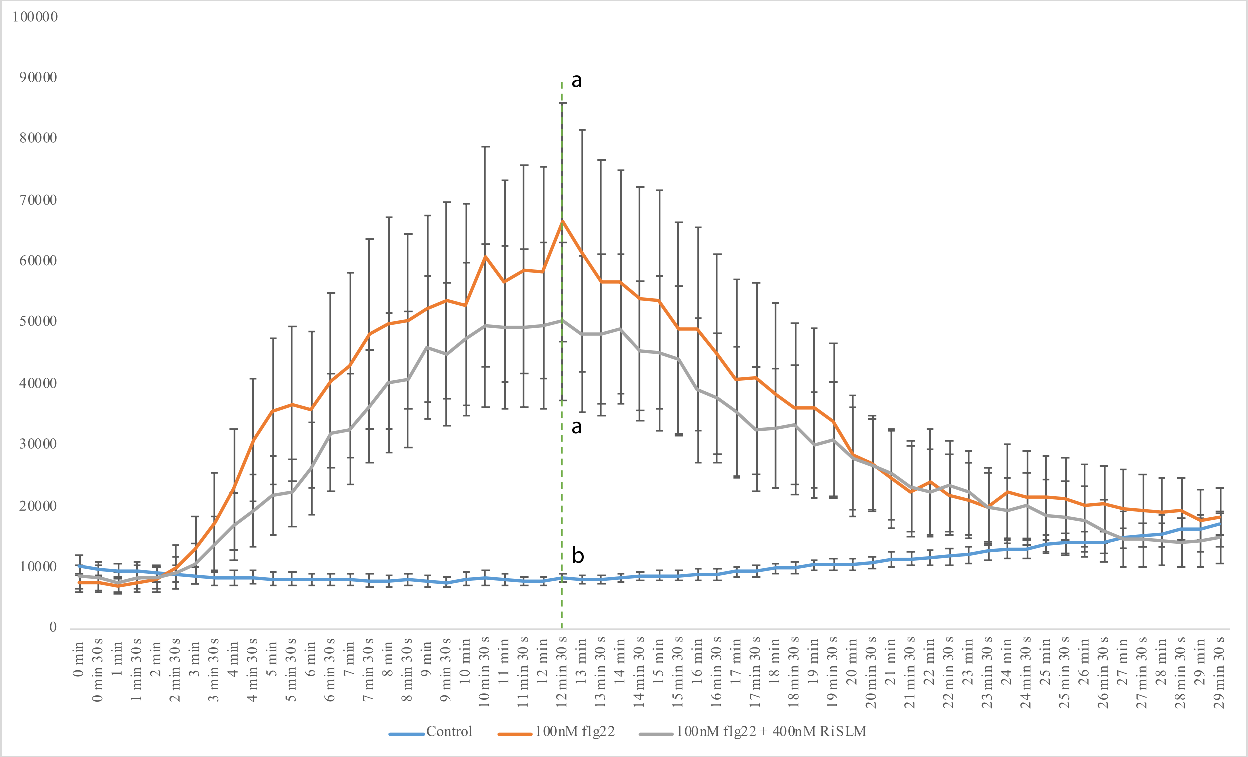

Fig. S6 RiSLM does not suppress flg22-induced reactive oxygen species (ROS) production. *Medicago truncatula* A17 root pieces were treated with 100nM flg22 with or without 400nM RiSLM . Error bars represent standard error from 6 (control and 100nM flg22 + 400nM RiSLM treated) or 5 (100nM flg22 treated) biological replicates. Different letters indicate significant difference (LSD $p<0.05$) between different treatments at the indicate time point (green dotted line).

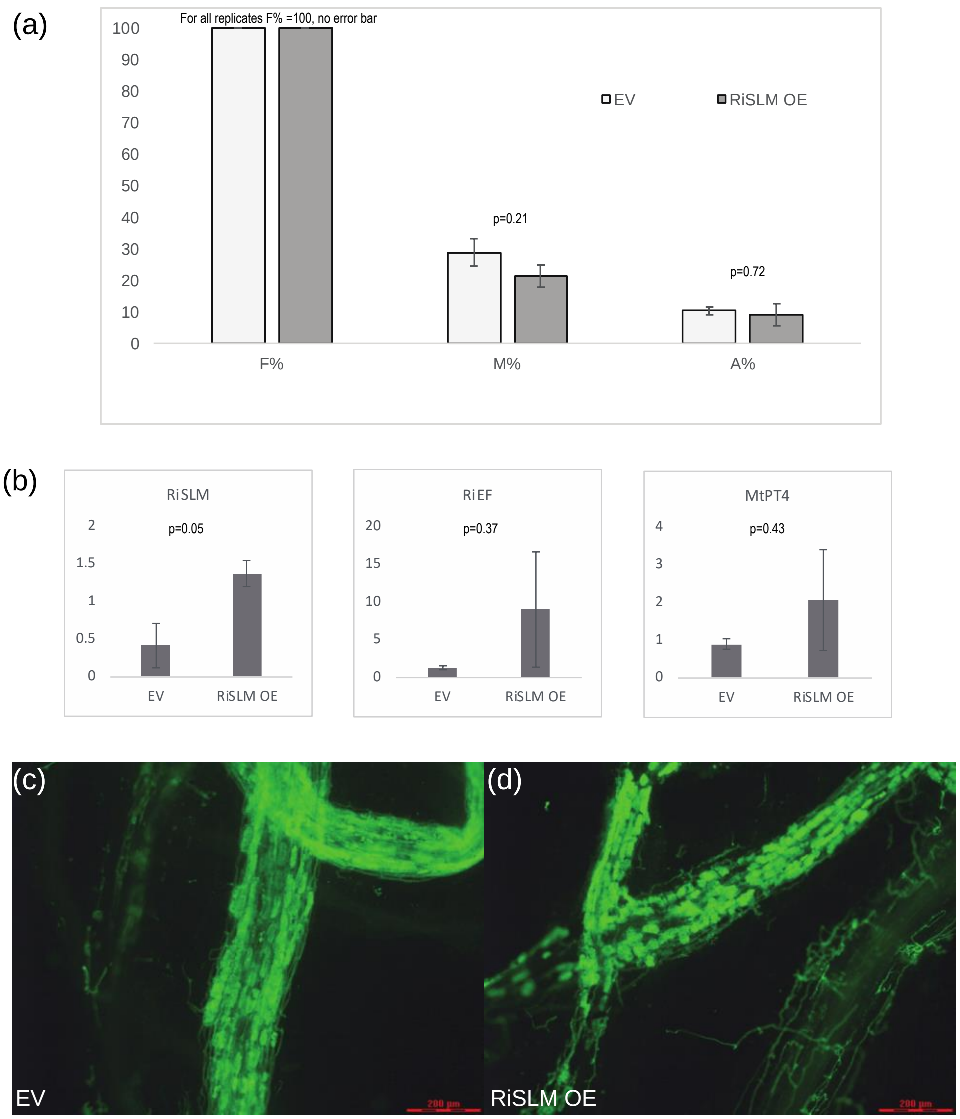

Fig. S7 Figure S7. *RiSLM* overexpression does not enhance mycorrhization in *Medicago truncatula*. (a) Frequency (F%), mycorrhization intensity in the root (M%), or arbuscule abundance in the root (A%) are not affected by overexpressing of *RiSLM*. (b) qPCR analysis of control and *RiSLM* overexpressing roots showing *RiSLM* expression level relative to *Rhizophagus irregularis* elongation factor *RiEF* and *MtPT4* expression levels relative to Medicago elongation factor *MtEF*. Error bar represents standard error from 3 replicates. (c, d) WGA-alexa488 staining of mycorrhization in *RiSLM* overexpressed roots (d) or control roots (c). Scale bar = 200µm.

Table S1 Primers used in this research.

Primer	Sequence	Note
RiSLM_SP-F	CTGAATTCATGAGACTCAATCAATCTTTGCTC	for cloning into pYST-02
RiSLM_SP-R	AAGCGGCCGCAAACATCTAGTAGCATTAGGTA TCC	
MtEPI-qPCRF	GGTTGTGTGTGTAACAGGTGCTTCA	qPCR primers for three defence marker genes
MtEPI-qPCRR	AGGGTCACGAACAGTGGCTCTA	
MtPAL-qPCRF	GCAAGGGCCGGTGTTGAAGC	
MtPAL-qPCRR	GGCAGCACCAAAGCCAGTGGT	
MtTHA-qPCRF	GGCGCAATCCCACCAGCAAC	
MtTHA-qPCRR	ACCACTCCCACCTTGTGGCG	
MtVapyrin-qPCRF	TCATCCTCCACAACAACAAGGT	qPCR primers for three symbiotic marker genes
MtVapyrin-qPCRR	TCAAGCACTTCTCTTATGTCATCCATTG	
MtPUB1-qPCRF	GTAGCAGCAAAAGGCACAAG	
MtPUB1-qPCRR	ATATCGGTCGAACCAGTTGC	
MtTUBB1-qPCRF	GAACAGTTCTCTGCCATGTTCAGAA	
MtTUBB1-qPCRR	TCTTCATAAACTGGGGCATCCTGGT	
MtEF-qPCRF	GATTGCCACACCTCTCACAT	qPCR primer for Medicago EF
MtEF-qPCRR	TCAGCGAAGGTCTCAACCAC	
RiEF-qPCRF	AACCCCTTCGTCTTCCACTT	qPCR primer for Rizophagus EF
RiEF-qPCRR	ATTGTTCGTGGTGCATTTCA	
RiSLM-qPCRF	AACGTAATCTTTCCCGGTTG	qPCR primer for RiSLM
RiSLM-qPCRR	TGGTGTATTTCTCTCACGGCTA	
RiSLM-HIGS-F	AAAAAAGCAGGCTTCATGAGACTCAATCAATCTTTG CTC	primers to make RiSLM HIGS construct
RiSLM-HIGS-R	CAAGAAAGCTGGGTCGATTTGTAATACAAGGATTTG CTCT	
RiSLM_F_EcoR1	GAATTCGCAATTAAGACATGTACTCC	primers used to clone RiSLM into pET-SUMO
RiSLM_R_Hind3	AAGCTTATTATTAAAAACATCTAGTAGCATTAG GTATCC	
F-RiSLM	AGAAGACTCAGGTGCAATTAAGACATGTACTC CG	Primers for L0 golden gate cloning (*RiSLM* overexpression)
R-RiSLM	GGAAGACAGCGAAAAACATCTAGTAGCATTAG G	

Table S2 Coding sequences of LysM effectors from different AM fungal species or isolates.

RiSLM [organism= *Rhizophagus irregularis*] isolate DAOM197198 LysM effector RiSLM completely CDS	ATGAGACTCAATCAATCTTTGCTCCTCTTAACCGTCCTTTTCG<br>CCCTTATTGCTGTTGCTTCATGTGCAATTAAGACATGTACTCC<br>GGTTTATGTTGTTGAGAGTGGTGATACCCTTGAAAAAATCGC<br>GAATAAACTTAAGGTGACCTTGCTAGTTTTGAAAAGAGCAAA<br>TCCTTGTATTACAAATCCGAACGTAATCTTTCCCGGTTGTATT<br>ATCCGGATACCTAATGCTACTAGATGTTTTTAA
RiSLM_A1 [organism= *Rhizophagus irregularis*] isolate A1 LysM effector completely CDS	ATGAGACTCAATCAATCTTTGCTCCTCTTAACCGTCCTTTTCG<br>CCCTTATTGCTGTTGCTTCATGTGCAATTAAGACATGTACTCC<br>GGTTTATGTTGTTGAGAGTGGTGATACCCTTGAAAAAATCGC<br>GAATAAACTTAAGGTGACCTTGCTAGTTTTGAAAAGAGCAAA<br>TCCTTGTATTACAAATCCGAACGTAATCTTTCCCGGTTGTATT<br>ATCCGGATACCTAATGCTACTAGATGTTTTTAA
RiSLM_A5 [organism= *Rhizophagus irregularis*] isolate A5 LysM effector completely CDS	ATGAGACTCAATCAATCTTTGCTCCTCTTAACCGTCCTTTTCG<br>CCCTTATTGCTGTTGCTTCATGTGCAATTAAGACATGTACTCC<br>GGTTTATGTTGTTGAGAGTGGTGATACCCTTGAAAAAATCGC<br>GAATAAACTTAAGGTGACCTTGCTAGTTTTGAAAAGAGCAAA<br>TCCTTGTATTACAAATCCGAACGTAATCTTTCCCGGTTGTATT<br>ATCCGGATACCTAATGCTACTAGATGTTTTTAA
RiSLM-1_A4 [organism= *Rhizophagus irregularis*] isolate A4 LysM effector-1 completely CDS	ATGAGACTCAATCAATCTTTGCTCCTCTTAACCATCCTTTTCG<br>CCCTTATTGCTGTTGCTTCATGTGCAGTTAAGACATGTACTCC<br>GGTTTATGTTGTTGAGAGTGGTGATACCCTTGATAAAATCGC<br>GAATAAACTTAAGGTGTCCTTGCCAGATTTGAAAAAAGCAAA<br>TCCTTGTATTACAAATCCGAACTTAATCTTTGTCGGTTGTATT<br>ATCCGGATACCTAATGCTACTGGATGTTTTTAA
RiSLM-2_A4 [organism= *Rhizophagus irregularis*] isolate A4 LysM effector-2 completely CDS	ATGAGACTCAATAAATCTTTGCTCCTCTTAACCATCCTTTTCG<br>CCCTTATTGCTGTTGCTTCATCTCAGCGTCTGACGACATGTAT<br>TCGGGTTTATATTGTTGTGCCTGGTGATACCCTTAATAAAATC<br>GCGATAAGTTTTGGTGTGTCCTTGAATGACTTGAAAAAAGCA<br>AATCCTTGTATTACAAATCCGAACTTAATCTTTCCCGGTTGCA<br>TTATCCGGATACCTAATCGTACTCAATGTTTTTAA
RiSLM-3_A4 [organism= *Rhizophagus irregularis*] isolate A4 LysM effector-3 completely CDS	ATGAGACTCAGCCTATTTTTTCTCCCCTTAACCATCATTTTCG<br>CCCTTATTGCTGCTGTTGCTTCGGCTGGTTGGTATATAGTTAA<br>ACAGGGTGATACCCTTTCTGCTATCGCGGATGGGTTTCATGTA<br>TCCTTGCAAGCTTTGGAAGCCGCTAATCCGGGAGTTACTCCG<br>GACAGAATCTTTCCTGGACAAGCTATCTGGATACCCTAA
RiSLM-4_A4 [organism= *Rhizophagus irregularis*] isolate A4 LysM effector-4 completely CDS	ATGAGACTCAGCCTATTTTTTCTCCCCTTAACCATCTTTTTCGC<br>CCTTATTGCTTCTGTTGCTTCGGCTGGTTGGTATATAGTTAGA<br>CAGGGTGATACCCTTTATGCTATCGCGAATGCGTTTCATATAT<br>CCTTGCAAGCTTTGGAAGCCGCTAATCCGGGAGTTACTCCGG<br>ACAGAATCTATCCTGGACAAGCTATCTGGATACCCTAA
RiSLM_B3	ATGAGACTCAATCTATCTTTGCTCCCCTTGACCATCCTTTTCG

[organism= *Rhizophagus irregularis*] isolate B3 LysM effector completely CDS	CCCTTATTGCTGTTGCTTCATGTGCAACTATGAAATGTGCTCC GGTTTATGTTGTTGAGGATAAAGATACTCTTGAAAAAATCGC GAAAAAACTTAAGGTGCCCTTGCCAGATTTGATAAAAGCTAA TCCTTGTATTTCAAATCCGGACGAAATCTATCCTGATTGCATG ATCCGGATACCTAAACAAACTAAATGCACTAAGGTTTGTATT TTATCTTTATTGTTTATATTTATCGATTACTACTAA
RiSLM_C2 [organism= *Rhizophagus irregularis*] isolate C2 LysM effector completely CDS	ATGAGACTCAATCAATCTTTGCTCCTCTTAACCATCCTTTTCG CCCTTATTGCTGTTGCATCATCTCAGCGTCTGACGACATGTAT TCAGGTTTATATTGTTGTGCCTGGTGATACCCTTAATAAAATC GCGATAAGTTTTGGTGTGTCCTTGAATGACTTGAAAAAAGCA AATCCTTGTATTACAAATCCGAACTTAATCTTTCCCGGTTGCA TTATCCGGATACCTAATCGTACTAAATGTTTTTAA
RcSLM [organism= *Rhizophagus clarus*] LysM effector completely CDS	ATGAAACACAATCTATTTTTGCTCTCCTTAACCATCCTTTTCG CGCTTAGTGCTTTTACTGATGTTACTTCGGCTGCTGCGAAAGG GTTTAGATATGTTGTTAAAAAGGGTGATACCCTTACCAGCAT CGCGAAAACTTTTAAAGTAAAATTGCCTGATTTGATCGCGGC TAATAAGCAGTGTGTTCCAAATCCGGACGTAATCTTTCCCAA ACAAGGTATCGCGATACCTCAGTACTGTCCCGTCTGTCCGTA G
GrSLM [organism= *Gigaspora rosea*] LysM effector completely CDS	ATGAGACTCTGTTTATTTTTGCTTCCCTTAACCATCATTTTCGC CTTTATTGCTGCTGTTGCTTCAATCAATTGTTGCTATACAGTT AACTCAGGCGATTCCCTTTATTCCATCGCAATTAATCATGGCA TAAGCTTGCAAGAAATGAAAGATGCTAATCCATGCATTAAAT ACCCGTACACTATTTATCCCAATCAAAAAATCACGCTCCCTA ACCAACCATACTGTTGA
GmSLM [organism= *Gigaspora margarita*] LysM effector completely CDS	ATGAGACTCAATCAATATTTGCTCCTCTTAACCGTCCTTTTCG CCCTTATTGCTGTTGCTTCATGTGCAATTAAGACATGTACTCC GGTTTATGTTGTTGAGAGTGGTGATACCCTTGAAAAAATCGC GAATAAACTTAAGGTGACCTTGCTAGTTTTGAAAAGAGCAAA TCCTTGTATTACAAATCCGAACGTAATCTTTCCCGGTTGTATT ATCCGGATACCTAATGCTACTAGATGTTTTTAA
